# Editorial: How Can Secretomics Help Unravel the Secrets of Plant-Microbe Interactions?

**DOI:** 10.3389/fpls.2016.01777

**Published:** 2016-11-30

**Authors:** Delphine Vincent, Kim M. Plummer, Peter S. Solomon, Marc-Henri Lebrun, Dominique Job, Maryam Rafiqi

**Affiliations:** ^1^Department of Economic Development, Jobs, Transport and Resources, AgriBio, La Trobe UniversityBundoora, VIC, Australia; ^2^Animal, Plant and Soil Sciences Department, AgriBio, La Trobe UniversityBundoora, VIC, Australia; ^3^Plant Sciences Division, Research School of Biology, The Australian National UniversityCanberra, ACT, Australia; ^4^Institut National de la Recherche Agronomique-AgroParisTech, UMR INRA1290, Biologie et Gestion des Risques en Agriculture - Champignons Pathogènes des PlantesThiverval-Grignon, France; ^5^Centre National de la Recherche-Scientifique, UMR5240 Centre Nationnal de la Recherche Scientifique/University Claude Bernard Lyon 1/INSA/Bayer CropScience Joint Laboratory, Bayer CropScienceLyon, France; ^6^Jodrell Laboratory, Royal Botanic GardensKew, London, UK

**Keywords:** secretome, secretomics, pathogenic fungi, extracellular proteins, virulence factors, protein effectors, host-fungi interactions, diseases

Secretomics describes the global study of proteins that are secreted by a cell, a tissue or an organism, and has recently emerged as a field for which interest is rapidly growing. The versatility of oomycetes, fungi, and bacteria allows them to associate with plants in many ways depending on whether they grow as a biotroph, hemibiotroph, necrotroph, or saprotroph. When interacting with a live organism, a microbe will invade its plant host and manipulate its metabolism either detrimentally if it is a pathogen or beneficially if it is a symbiont. Deciphering secretomes became a crucial biological question when an increasing body of evidence indicated that secreted proteins were the main effectors initiating interactions, whether of pathogenic or symbiotic nature, between microbes and their plant hosts.

This Research Topic aims at discussing how secretomics can assist scientists in gaining knowledge about the mechanisms underpinning plant-microbe interactions. The following aspects are discussed.

## *In vitro* secretomics

González-Fernández et al. made an overview of the proteomics contribution to the study and knowledge of the extracellular secreted proteins of the fungal phytopathogen *Botrytis cinerea*. They hypothesized on the putative functions of these secreted proteins, and their connection to the biology of the *B. cinerea* interaction with its hosts.

Dieryckx et al. analyzed the inhibitory effect of salicylic acid (SA), a major plant hormone, on *in vitro* growth of *B. cinerea*. Comparative proteomics of intracellular and secreted proteomes revealed several mechanisms that could potentially account for the observed growth inhibition, notably pH regulation, metal homeostasis, mitochondrial respiration, ROS accumulation and cell wall remodeling.

Félix et al. explored the interesting case of a plant fungal pathogen *Lasiodiplodia theobromae* which evolves into a human pathogen strain when exposed to human body temperature. They show that both strains are cytotoxic to mammalian cells but while the environmental strain CAA019 is cytotoxic mainly at 25°C, the clinical strain CBS339.90 is cytotoxic mainly at 30 and 37°C. They demonstrate that temperature modulates the secretome of *L. theobromae*, which may be associated with host-specificity requirements.

## Proteases

Karimi Jashni et al. reviewed the recent advances on proteases and protease inhibitors (PIs) involved in fungal virulence and plant defense. They show that proteases and PIs from plants and their fungal pathogens play an important role in the arms race between plants and pathogens, which has resulted in co-evolutionary diversification and adaptation shaping pathogen lifestyles.

Lowe et al. used a systems biology approach comprising genome analysis, transcriptomics, and label-free quantitative proteomics to characterize peptidases deployed by the cereal pathogen *Fusarium graminearum* (Fgr) during growth. A high resolution mass spectrometry-based proteomics analysis defined the extracellular proteases secreted by Fgr. A meta-classification based on sequence features and transcriptional/translational activity *in planta* and *in vitro* provides a platform to develop control strategies that target Fgr peptidases.

## Bioinformatics and prediction tools

Sperschneider et al. assessed several secretion prediction tools on experimentally validated fungal and oomycete effectors. For a set of fungal SwissProt protein sequences, SignalP 4 and the neural network predictors of SignalP 3 (D-score) and SignalP 2 perform best. Yet, assessment of subcellular localization predictors indicates that effectors targeted to the host cytoplasm are often predicted as being not extracellular. This limits the reliability of secretion predictions that depend on these tools.

Kim et al. identified small secreted proteins (SSPs) in 136 fungal species from data archived in the Fungal Secretome Database via a refined secretome workflow. They observed that species that are intimately associated with host cells, such as biotrophs and symbionts, usually have higher proportion of species-specific SSPs (SSSPs) than hemibiotrophs and necrotrophs, but the latter groups displayed higher proportions of secreted enzymes.

Badet et al. analyzed amino acids usage and intrinsic protein disorder in alignments of groups of orthologous proteins from three *Sclerotiniaceae* species. Enrichment in Thr, depletion in Glu and Lys, and low disorder frequency in hot loops are significantly associated with proteins from *S. borealis*. The results also highlight a novel putative antifreeze protein and a novel putative lytic polysaccharide monooxygenase.

## Exosomes

Samuel et al. discussed current knowledge on extracellular vesicles (EVs) in the context of human-fungal interactions and their potential roles in plant-fungal interactions. They propose that the molecular cargo present in EVs is specific to the type of insult or infection.

## Effectors

Canker caused by the Ascomycete *Valsa mali* is the most destructive disease of apple in Eastern Asia. Li et al. identified and characterized the *V. mali* repertoire of candidate effector proteins (CEPs). Based on transient over-expression in *Nicotiana benthamiana* performed for 70 randomly selected CEPs, seven of them were shown to significantly suppress BAX-induced programmed cell death. Furthermore, targeted deletion of the genes encoding these proteins resulted in a significant reduction of virulence.

Mesarich et al. reviewed the diverse roles of several effectors of plant-associated organisms corresponding to repeat-containing proteins (RCPs) that carry tandem or non-tandem arrays of an amino acid sequence or structural motif. This analysis draws attention to the potential role of these repeat domains in adaptive evolution with regards to RCP effector function and the evasion of effector-triggered immunity.

Lorrain et al. reviewed the current status of the poplar rust fungus secretome and prediction of candidate effectors from this species. They stress that effector mining in the poplar rust fungus relies both on the quality of input data (i.e., gene annotation and gene family analyses) and on several qualitative and subjective criteria.

Tan et al. characterized three protein effectors, namely SnToxA, SnTox1, and SnTox3, which are involved in *Septoria nodorum* blotch (*Parastagonospora nodorum*). From deletion analyses they conclude that the secreted necrotrophic effectors explain a very large part of the disease response of wheat germplasm and that this method of resistance breeding promises to further reduce the impact of this devastating disease.

## Toward breeding resistance to pathogens in crops

Breen et al. reviewed the present knowledge on antimicrobial peptides (AMPs) that are involved in the innate immune system against pathogen attacks. They highlight that such AMPs could offer a solution to combat microbial disease in crops by exploring not only the plant-derived AMPs, but also non-plant AMPs produced by bacteria, fungi, oomycetes, or animals. The greatest challenge remains the functional validation of candidate AMPs *in planta* through transgenic experiments, particularly introducing AMPs into crops.

*AvrLm6-like* genes are present as large families (>15 members) in all sequenced strains of *Venturia inaequalis* (apple scab pathogen) and *V. pirina* (European pear scab pathogen) (Shiller et al.). These genes are located in gene-poor regions of the genomes, and mostly in close proximity to transposable elements, which may explain the expansion of these gene families. An *AvrLm6* homolog from *V. inaequalis* that is up-regulated during infection was shown to be localized to the sub-cuticular stroma during biotrophic infection of apple hypocotyls.

## Conclusion

Publications in this Research Topic highlight the range of activities currently being undertaken by microbial secretomics researchers and reflect the current state of the field. This topic is still in its infancy and much remains to be accomplished at the experimental level (e.g., preparation of the secretomes, methodologies of secretomics, mechanisms of protein secretion, functional validation of the secreted proteins). The findings reported here are however very encouraging as they emphasize the major roles of microbial secretomes enabling interactions with plant hosts. It is anticipated that this knowledge will be useful for characterizing genes encoding secreted proteins as novel targets for crop breeding.

## Author contributions

DJ wrote the draft. DV, KP, PS, ML, and MR edited and contributed to the initial draft. DV compiled, formatted, and submitted the final version.

### Conflict of interest statement

The authors declare that the research was conducted in the absence of any commercial or financial relationships that could be construed as a potential conflict of interest.

